# The Relationship Between Vertical Facial Type and Maxillary Anterior Alveolar Angle in Adults Using Cone-Beam Computed Tomography

**DOI:** 10.7759/cureus.30356

**Published:** 2022-10-16

**Authors:** Alia Abdul-Sattar El-Schallah, Mowaffak A Ajaj, Mohammad Y Hajeer

**Affiliations:** 1 Department of Orthodontics, Damascus University Faculty of Dentistry, Damascus, SYR

**Keywords:** hyperdivergent, normodivergent, hypodivergent, maxillary anterior alveolar angle, cbct, vertical facial type

## Abstract

Background

Cone-beam computed tomography (CBCT) imaging provides detailed and thorough information about the dentofacial complex. However, not all aspects have been yet explored among different types of malocclusion. The maxillary anterior alveolus is one of the components of the maxillary bone which affects the upper lip position and the esthetics of the smile. The inclination of this alveolus may vary between the different vertical growth patterns of patients who may seek orthodontic treatment. The objective of this study was to investigate possible differences in maxillary anterior alveolar angle (MAAA) among orthodontically untreated adults with different vertical facial types in a Syrian sample.

Methods

CBCT images of 84 orthodontically untreated adult patients were included. Three groups of vertical facial type (n=28 for each group; 14 males, 14 females) were created using disproportionate multi-stratified random sampling. CBCT-derived lateral cephalograms were used to categorize the patients into three groups. Measurements were made at three regions (region 1 (R1), region 2 (R2), and region 3 (R3)), located in the maxillary anterior alveolar bone using OnDemand3D™ software (Cypermed Inc., Seoul, South Korea).

Results

No significant differences in the mean MAAA were detected between females and males for the three measured regions in all groups. Analysis of variance showed significant inter-group differences in the MAAA (p<0.05) for all measured regions. The hyperdivergent facial type group had the greatest MAAA mean value of 68.72° (± 6.01), 67.30° (± 4.15), and 68.01° (± 5.12) at R1 in the female, male, and the entire sample of both sexes respectively. Whereas the hypodivergent facial type group had the least mean MAAA values of 58.47° (± 5.34) at R3, 59.83° (± 6.23) at R2, and 59.23° (± 5.75) at R3 in the female, male, and the entire sample of both sexes respectively.

Conclusions

The maxillary anterior alveolar bone was more buccally inclined in the hypodivergent facial type. The MAA bone inclination did not differ between females and males in the same vertical facial type group.

## Introduction

Craniofacial morphology is mainly determined by genetic factors, although functional demands have a significant effect on its growth and development [1]. Frost’s mechanistic hypothesis explained bone adaptation to masticatory muscle force as a type of adaptation to functional demands [2-6]. Frost has suggested that there is a range of strains maintaining the form and mass of bone, and he explained that strains below or above this range induce changes in this bone characteristics [5,6]. Since muscles exert forces that strain the bone, lighter forces might induce less cortical bone adaptations [6]. It has been concluded that there is a relationship between facial divergence and masticatory muscle function [7]. Recent studies using cone-beam computed tomography (CBCT) have shown differences in maxillary and mandibular cortical bone thickness, height, density, and buccal cortical plate inclination among patients with different vertical facial types [6, 8-13]. These differences were classified as a type of compensatory mechanism [14]. This dentoalveolar compensatory mechanism could be defined as a system that attempts to maintain a normal or optimal interdigitation of interdental and interalveolar arch relations under varying jaw relationships and facial types [15]. Studies indicated that these dissimilarities in quality and quantity of the dentoalveolar process among vertical facial types are parameters relevant to the success of temporary skeletal anchorage devices (TSADs) in promoting absolute anchorage [11,16,17].

The simple and easy placement and removal, small size, cost efficiency, and convenience for achieving a reliable source of anchorage have recently made TSADs very popular in orthodontic treatment [18]. Primary factors for TSADs' stability are alveolar bone quality, cortical bone thickness, implant design, insertion method, and the insertion angle [19-22].

The vertical facial type is one of the principal components of deciding the most appropriate treatment plan for orthodontic patients [23]. Considering the variance in cortical bone characteristics among the three vertical facial types, and approaching it as a modification in the limitations of orthodontic tooth movement within the alveolar bone envelope “orthodontic wall” as it was defined by Handelman is important, because challenging these boundaries may lead to to iatrogenic sequelae [24]. Furthermore, extraction decisions, treatment preparations, treatment mechanics, retention period, and type of anchorage are all influenced by the vertical growth pattern [25].

The use of CBCT has increased in popularity in orthodontics [26-29]. Recently, studies have investigated differences in quantitative, qualitative, and morphological characteristics of the maxilla and mandible in different vertical facial types [6,10-12,23], but only a few analyzed the differences in these characteristics between the genders in these types. Although the maxillary cortical bone inclination of the alveolar process has been studied in the posterior buccal region using CBCT in adult patients with different vertical facial types [12], the inclination of the maxillary anterior alveolar bone in these vertical facial types has not been evaluated yet.

Therefore, the purpose of this study was to evaluate whether the inclination of the maxillary anterior alveolar bone varied among patients with hypodivergent, normodivergent, and hyperdivergent facial types in a Syrian sample and to determine whether gender dimorphism existed among those patients using CBCT images.

## Materials and methods

Study design, CBCT collection, inclusion criteria

**Figure 1:**
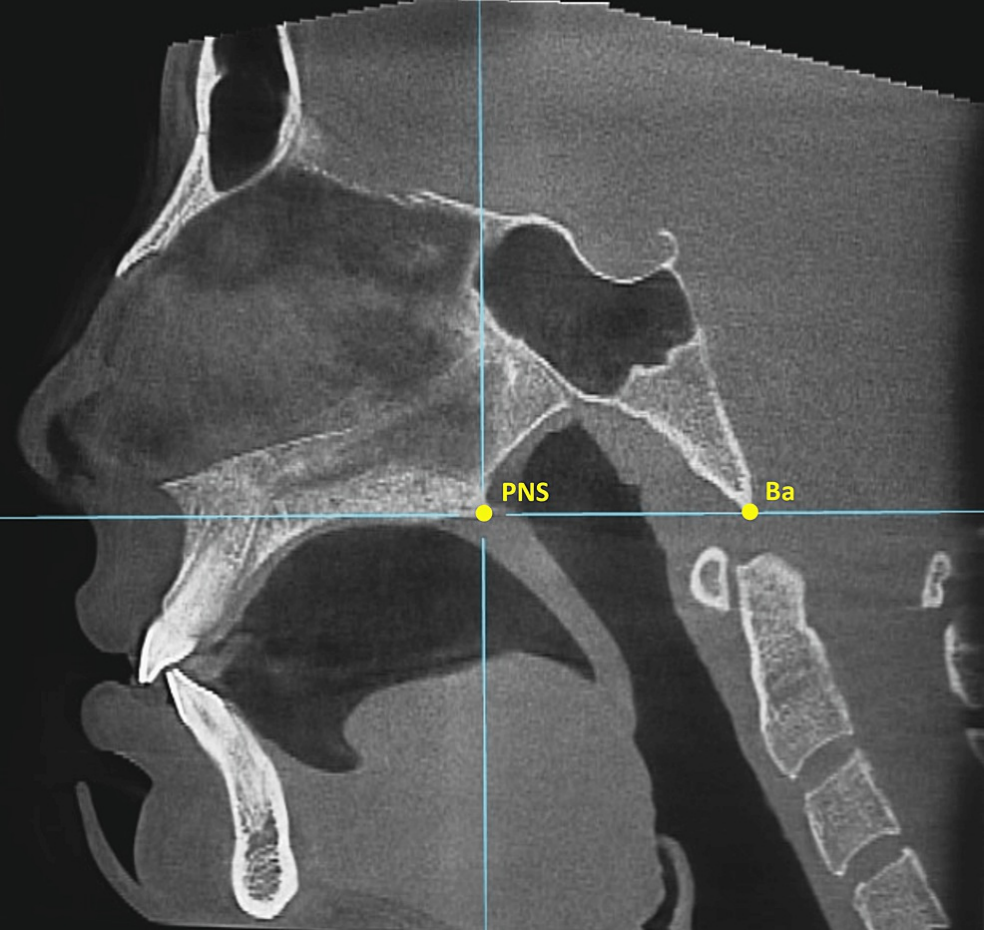
The head's orientation in the sagittal view according to the posterior nasal spine (PNS) and the basion point (Ba).

**Figure 2:**
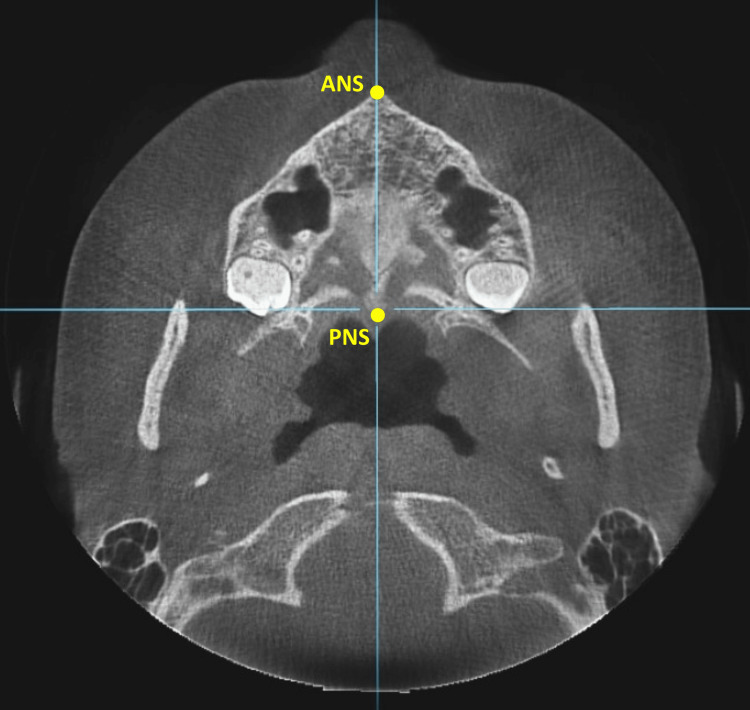
The head's orientation on the axial view according to the anterior nasal spine (ANS) and the posterior nasal spine (PNS).

The study sample was selected by retrospective screening of CBCT images archived at the Department of Orthodontics, University of Damascus Dental School, Syria. A disproportionate multi-stratified random sampling from a sampling frame of 540 patients was employed to assign the patients into the three groups. CBCT images were collected in the period of the past five years. CBCT images of these subjects had been taken for several reasons, such as pre-extraction confirmation of the status of teeth roots, planning orthognathic surgery cases, or assessment of impacted teeth; therefore, they were not unnecessarily subjected to additional radiation. Subjects’ rights were protected, and approval was obtained from the Local Research Ethics Committee of the University of Damascus Dental School (UDDS-192-05022018/SRC-267). CBCT images were scanned with the imaging apparatus: SCANORA® 3DxDevice (Soredex, Tuusula, Finland) with 15 mA, 85 kV, 40-second exposure time, and isotropic voxel size of 0.25x0.25x0.25 mm. Files were saved as digital imaging and communications in medicine (DICOM) file format, and the images were viewed through OnDemand 3D™ software (CyberMed, Inc., Seoul, Korea). The CBCT images of 84 patients of both genders, 18 to 25 years of the same ethnic origin, were analyzed for this study. The CBCT images included the entire maxillofacial region, with fully erupted permanent maxillary central and lateral incisors and molars in contact during occlusion. The exclusion criteria were as follows: (1) previous or current orthodontic treatment, (2) periapical or periradicular pathologies or radiolucencies of either periodontal or endodontic origin or large metal restorations in the region to be studied, (3) severe facial or dental asymmetries, (4) evidence of previous trauma, crowding of the permanent maxillary central and lateral incisors. Only highly reproducible hard-tissue landmarks were used in this study. The employed landmarks were Basion (Ba), the posterior nasal spine (PNS), and the anterior nasal spine (ANS) [30]. All CBCT images were oriented (adjusted by 2D rotation of the slice) so that the horizontal axis passed through Ba posteriorly and PNS anteriorly in the sagittal view (Figure 1), and the axial axis passed through ANS and PNS in the axial view (Figure 2). This orientation was performed to minimize the measurement errors produced by non-standardized head postures. The subjects were classified into three groups based on their vertical facial pattern; hypodivergent, normodivergent, and hypodivergent facial. The group categories were determined on CBCT-derived lateral cephalograms using the maximum intensity projection (MIP) technique [31].

Categories were determined using one linear and one angular measurement: the facial height index (FHI) [32] and the Frankfort mandibular plane angle (FMA) [33], as shown in Table 1. Patients had to fit into a single category for both variables to be included in that specific category in the sampling frame.

**Table 1:** Values of the facial height index (FHI) and the Frankfort mandibular plane angle (FMA) used to determine the vertical facial type of the patients included in the study.

Variable	Hypodivergent	Normodivergent	Hyperdivergent
Facial Height Index	> 69%	61% - 69%	< 61%
Frankfort Mandibular Plane Angle	< 21º	29º - 21º	> 29º

Maxillary anterior alveolar angle measurement 

**Figure 3:**
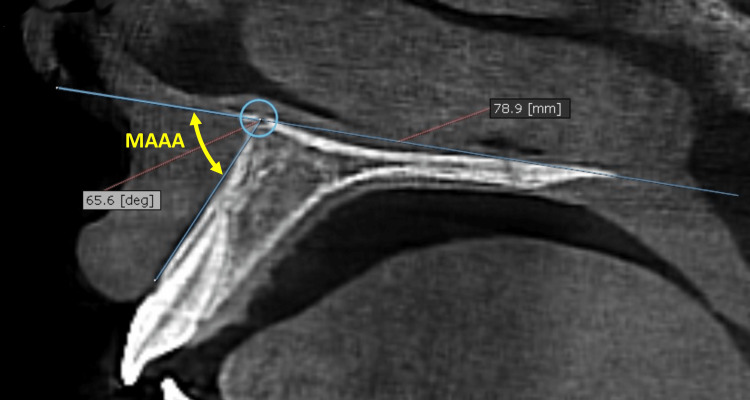
The MAAA on the sagittal view depends on measuring the outer angle created by the intersection between the palatal plane and a line tangent to the anterior alveolus MAAA: maxillary anterior alveolar angle

**Figure 4:**
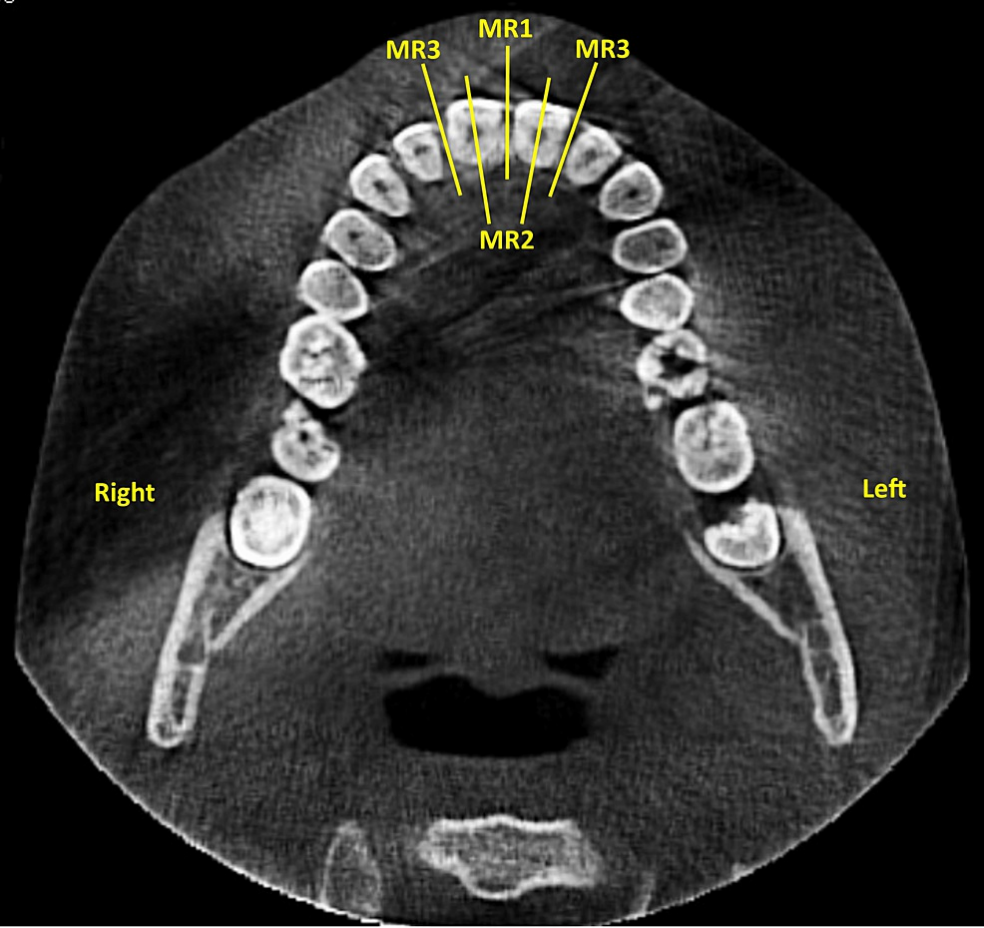
The measuring regions on the axial view for the maxillary anterior alveolar angle. MR1: The region bisecting the interdental space between the two permanent maxillary central incisors; MR2: The region bisecting the root of the permanent maxillary central incisor; MR3: The region bisecting the interdental space between the permanent maxillary central and lateral incisors.

Sagittal slices of each CBCT image were obtained in three regions to measure the maxillary anterior alveolar angle (MAAA). MAAA was defined as: The outer angle created between the palatal plane (ANS-PNS) and a line tangent to the anterior alveolus, measured on the sagittal view of a CBCT image (Figure 3). The axial slice was adapted for each one of the three regions to be measured: region 1 (R1), region 2 (R2), and Region 3 (R3) as follows: (1) R1: On the axial view, the vertical axis was oriented so to bisect the interdental space between the two permanent maxillary central incisors (Figure 4); (2) R2: On the axial view, the vertical axis was oriented to bisect the root of the permanent maxillary central incisor; (3) R3: On the axial view, the vertical axis was oriented so to bisect the interdental space between permanent maxillary central and lateral incisors.

 

Statistical analysis

All statistical analyses were performed using IBM SPSS Statistics for Windows, Version 20.0 (Released 2011; IBM Corp., Armonk, New York, United States). Normality tests showed normal distribution of the gathered data. Therefore, parametric tests were employed. Descriptive statistics are presented in (Table 3). MAAA in the regions R2 and R3 was measured for the right and left sides. Paired t-tests revealed that there were no significant differences between both sides. Therefore, the mean values of right and left measurements were used. Independent t-tests were used to evaluate differences in the MAAA between females and males in each vertical facial type group. One-way ANOVA was used to detect significant differences between the three groups. The level of significance was set at 5%.

Methodological error

To determine systematic and random errors, 10 CBCT images were randomly selected after a four-week interval and were re-measured by the same observer (A.A.S), who had been trained in reading and measuring CBCT images for four years. Paired t-test and reliability test to calculate the intraclass correlation coefficient (ICCs) were performed.

## Results

Basic sample characteristics

The basic sample characteristics are given in Table 2. The mean age in the six created subgroups (according to the facial vertical pattern and gender) ranged from 20.0 to 21.5 years. The mean facial height index and the mean Frankfort mandibular plane angle conformed to the criteria employed to create the three subgroups based on the vertical dimension.

**Table 2:** Descriptive statistics of the facial height index, the Frankfort mandibular plane angle, and age according to the vertical facial type of the patients included in the study.

	Hypodivergent		Normodivergent		Hyperdivergent	
Variables	Female (n=14)	Male (n=14)	Female (n=14)	Male (n=14)	Female (n=14)	Male (n=14)
Age (year)	21.0±1.9	20.6±2.1	20.7±1.7	21.3±2.0	20.5±1.7	21.5±2.1
FHI	72.0±2.3	73.2±4.0	63.7±1.5	64.6±1.8	57.7±2.8	57.0±5.0
FMA	14.9±3.4	14.7±4.0	24.0±1.1	23.8±1.9	31.6±2.5	31.3±2.6

The error of the method

No systematic error was found between the first and second measurements for all the measured variables. All ICC values were greater than 0.95, indicating high intra-observer reliability (Table 3).

**Table 3:** Assessment of the intra-observer reliability and error of the method *ICC: Intraclass correlation coefficients; **p-value of paired t-tests MR1: measuring region 1; MR2: measuring region 2; MR3: measuring region 3.

Variable	First measurement		Second measurement		ICC^*^	Mean difference	p-value^**^
	Mean	SD	Mean	SD			
MR1	63.58	3.93	63.52	4.45	0.985	0.06	0.845
MR2	63.25	3.08	63	2.75	0.973	0.25	0.425
MR3	62.97	3.64	63.13	3.76	0.992	-0.16	0.992

Main findings

Descriptive statistics of MAAA for both genders and the entire sample of the three vertical facial type groups are given in Table 4.

**Table 4:** Descriptive statistics of maxillary anterior alveolar angle (MAAA) for both genders and the entire sample of the three vertical facial type groups MR1: The region bisecting the interdental space between the two permanent maxillary central incisors; MR2: The region bisecting the root of the permanent maxillary central incisor; MR3: The region bisecting the interdental space between the permanent maxillary central and lateral incisors.

Group	Region	Females		Males		Entire Sample	
		Mean	SD	Mean	SD	Mean	SD
Hypodivergent	MR1	61.73	5.67	60.90	4.38	61.32	4.99
	MR2	59.97	4.65	59.83	6.23	59.90	5.39
	MR3	58.47	5.34	60.00	6.24	59.23	5.75
Normodivergent	MR1	65.43	3.57	63.11	4.08	64.27	3.94
	MR2	63.01	5.19	61.00	4.37	62.01	4.81
	MR3	61.33	5.55	60.82	4.16	61.08	4.82
Hyperdivergent	MR1	68.72	6.01	67.30	4.15	68.01	5.12
	MR2	65.68	7.09	66.61	4.93	66.15	6.01
	MR3	64.70	6.24	66.37	3.85	65.53	5.16

No statistically significant differences were found between females and males in the three vertical facial type groups (Table 5). Statistically significant differences were found when comparing the three vertical facial type groups in female, male, and the entire sample; in both genders and the entire sample, the mean values for all three regions were greatest in the hyperdivergent facial type group, and least in the hypodivergent facial type group, with the normodivergent facial type group having an intermediate value between both of them.

**Table 5:** The differences in the maxillary anterior alveolar angle (MAAA) between females and males in the three vertical facial type groups. MR1: The region bisecting the interdental space between the two permanent maxillary central incisors; MR2: The region bisecting the root of the permanent maxillary central incisor; MR3: The region bisecting the interdental space between the permanent maxillary central and lateral incisors.

Group	Region	Female	Male	P-value
		Mean±SD	Mean±SD	
Hypodivergent	MR1	61.73±5.67	60.90±4.38	0.669
	MR2	59.97±4.65	59.83±6.23	0.948
	MR3	58.47±5.34	60.00±6.24	0.495
Normodivergent	MR1	65.43±3.57	63.11±4.08	0.121
	MR2	63.01±5.19	61.00±4.37	0.279
	MR3	61.33±5.55	60.82±4.16	0.787
Hyperdivergent	MR1	68.72±6.01	67.30±4.15	0.472
	MR2	65.68±7.09	66.61±4.93	0.692
	MR3	64.70±6.24	66.37±3.85	0.400

Post-hoc tests revealed significant differences between the hyperdivergent and the hypodivergent facial type groups and between the hyperdivergent and the normodivergent facial type groups in male patients and the entire sample. In female patients, post-hoc tests revealed significant differences between the hyperdivergent and the hypodivergent facial type groups, as shown in Table 6.

**Table 6:** The results of the analysis of variance (ANOVA) and post-hoc tests of the maxillary anterior alveolar angle (MAAA) in females, males, and the entire sample for the three vertical facial type groups * Differences with P < 0.05, ** differences with P < 0.01. MR1: The region bisecting the interdental space between the two permanent maxillary central incisors; MR2: The region bisecting the root of the permanent maxillary central incisor; MR3: The region bisecting the interdental space between the permanent maxillary central and lateral incisors; Hyper.:Hyperdivergent; Normo.:Normodivergent; Hypo.:Hypodivergent

		Hypodivergent	Normodivergent	Hyperdivergent	P Value	Tukey’s post-hoc tests	
Gender	Region	Mean±SD	Mean±SD	Mean±SD		Pairwise comparisons	Sig.
Female	MR1	61.73±5.67	65.43±3.57	68.72±6.01	0.004**	Hyper. vs. Hypo.	0.003**
	MR2	59.97±4.65	63.01±5.19	65.68±7.09	0.041*	Hyper. vs. Hypo.	0.032*
	MR3	58.47±5.34	61.33±5.55	64.70±6.24	0.023*	Hyper. vs. Hypo.	0.018*
Male	MR1	60.90±4.38	63.11±4.08	67.30±4.15	0.001**	Hyper. vs. Hypo.	0.001**
						Hyper. vs. Normo.	0.032*
	MR2	59.83±6.23	61.00±4.37	66.61±4.93	0.003**	Hyper. vs. Hypo.	0.004**
						Hyper. vs. Normo.	0.019*
	MR3	60.00±6.24	60.82±4.16	66.37±3.85	0.002**	Hyper. vs. Hypo.	0.004**
						Hyper. vs. Normo.	0.012*
Entire Sample	MR1	61.32±4.99	64.27±3.94	68.01±5.12	0.000**	Hyper. vs. Hypo.	0.000**
						Hyper. vs. Normo.	0.011*
	MR2	59.90±5.39	62.01±4.81	66.15±6.01	0.000**	Hyper. vs. Hypo.	0.000**
						Hyper. vs. Normo.	0.015*
	MR3	59.23±5.75	61.08±4.82	65.53±5.16	0.000**	Hyper. vs. Hypo.	0.000**
						Hyper. vs. Normo.	0.006**

## Discussion

This study compared the inclination of the maxillary anterior alveolar bone relative to the palatal plane among patients with different vertical facial types using CBCT images. The results of the present study showed that there were no significant differences in the mean MAAA values between females and males of the same vertical facial type. Still, there were significant differences in the mean MAAA values between different vertical facial types, with the hypodivergent group having the most buccally inclined maxillary anterior alveolar bone plates. In contrast, the hyperdivergent group had the most vertically oriented maxillary anterior alveolar bone plates. To the best of our knowledge, this study is the first to assess the inclination of the upper anterior alveolar bone using CBCT images. Therefore, direct comparison with other studies conducted on other races or ethnicities is impossible.

The mean MAAA in the hyperdivergent group for the three measured regions (R1, R2, R3) was 6.41 degrees greater than in the hypodivergent group; the same pattern of difference has been previously shown in a study of the maxillary buccal cortical plate inclination but in the posterior regions [12]. Differences in the anterior alveolar bone inclination might be explained as dentoalveolar compensation. Dentoalveolar compensation is associated with the size, orientation, and strength of masticatory muscles, affecting facial divergence and maxillofacial complex development [34, 35]. Hyperdivergency has been linked to weak masticatory forces and muscular hypofunction [7,36]. Weakened masticatory muscles produce less force and, therefore, less strain on the bones to which they are attached [37-40] and, therefore, weaker buccally pushing masticatory forces, narrower dental arches, and more vertically oriented alveolar bone, thereby evoking a greater MAAA value. On the other side, heavier masticatory forces associated with the hypodivergent facial type increase the buccal inclination of the alveolar bone, giving the dental arch a broader appearance [41,42] and leading to a more buccally inclined maxillary anterior alveolar bone.

Moreover, a previous study by Masumoto et al. indicated that in hypodivergent subjects, the mandibular central incisors and the mandibular alveolar bone in the second molar region had the greatest amount of buccal inclination compared to the hyperdivergent facial type; this was explained as a result of increased masticatory muscle and bite force [9]. Facial divergence also had an impact on the buccolingual inclination of the molars, as Tsunory et al. and Masumoto et al. revealed that the hyperdivergent facial type had more upright posterior teeth than the hypodivergent facial type [9,43,44]; and this was found to be due to the reduced masticatory muscle force [43,45].

Other alveolar bone characteristics were also interpreted as dentoalveolar compensation. The increased alveolar bone height in the incisor region of hyperdivergent subjects was explained as compensation for the increased lower anterior facial height in this kind of facial growth pattern [8,44,46]. In addition, alveolar bone density and thickness have been explained as compensation related to masticatory muscle function. Reduced muscle force influences the amount of cortical and trabecular bone by reducing its amount in the dentoalveolar process [37,47]. Many previous reports have highlighted the presence of several dentoalveolar compensations with the hyperdivergent facial type in comparison with the hypodivergent facial type, and they commonly include: more buccally inclined alveolar bone plates [11], greater alveolar bone density [11,37,47], less alveolar bone height and thicker alveolar bone (width) [6,23], which all have to be taken into consideration when an orthodontic treatment plan is designed.

Because alveolar bone properties are the determinants of orthodontic tooth movement boundaries [24], tooth movement needs to be carried out carefully with a focus on its morphological and characteristical features associated with the vertical facial type, bearing in mind alveolar bone thickness when buccolingual bodily tooth movement is to be accomplished, or alveolar bone height, when intrusion is considered, or alveolar bone inclination, when buccolingual tipping is intended. Tozlu et al. recommended that the vertical facial type be considered when adjusting the insertion angle of TSADs in the maxillary buccal posterior regions [12].

TSADs that are inclined concerning the bone surface were found to create greater contact with the cortical bone, increasing the stability and mechanical retention of TSADs [17,21,48,49]. Petrey et al. demonstrated that the most effective TSAD insertion angle to the bone surface is 90º [50]. However, an angle such as 30º would grant a greater TSADs-bone surface contact. Nevertheless, it has the least insertion torque (IT), which is not recommended [21].

The current findings may also guide the use of TSADs in the maxillary anterior regions. When adjusting the insertion angle of a miniscrew, a more obliquely inserted miniscrew influences the IT and may expose a greater lever arm and a higher rate of failure [51]. An increased insertion angulation could cause slippage of the miniscrew at its first contact with bone [21]. In subjects with different vertical facial patterns, the unified use of a certain direction of penetration of miniscrews would produce a greater or less angle of insertion compared to that of the normodivergent facial type. The insertion angle is important to achieve a balanced amount of contact between the cortical bone, trabecular bone, and the TSAD surface [11]. Therefore, a more inclined insertion angle may lead to more contact with the cortical bone. With a more vertical insertion angle in subjects with hyperdivergent facial types, who already have thinner cortical bone, less contact with cortical bone is achieved, thereby increasing the risk of TSAD failure. When increased contact with cortical bone in the hypodivergent facial type is achieved, the risk of causing damage when inserting the TSAD, in the form of cracking or overheating the cortical bone, is higher, as this vertical facial type has a thicker and denser cortical bone.

Limitations

This study was conducted with a relatively small sample size of patients and on a specific ethnicity; therefore, it would be interesting to see reports from different countries representing different races with many patients. Finally, a study evaluating the possible correlation between alveolar bone inclination and masticatory muscle activity in both genders and different vertical facial types should also be considered.

## Conclusions

MAAA is greater in hyperdivergent facial type than in normodivergent and hypodivergent facial type subjects, with the normodivergent facial type group having an intermediate value between them. MAAA does not differ between female and male patients in the same vertical facial type group. Vertical facial type should be considered when planning orthodontic treatment or when temporary skeletal anchorage devices are inserted for anchorage.
